# IFNγ-Producing γ/δ T Cells Accumulate in the Fetal Brain Following Intrauterine Inflammation

**DOI:** 10.3389/fimmu.2021.741518

**Published:** 2021-10-04

**Authors:** Emma L. Lewis, Natalia Tulina, Lauren Anton, Amy G. Brown, Paige M. Porrett, Michal A. Elovitz

**Affiliations:** ^1^ Center for Research on Reproduction and Women’s Health, University of Pennsylvania, Philadelphia, PA, United States; ^2^ Department of Surgery, Perelman School of Medicine, University of Pennsylvania, PA, United States; ^3^ Department of Obstetrics and Gynecology, Perelman School of Medicine, University of Pennsylvania, Philadelphia, PA, United States

**Keywords:** maternal-fetal interface, fetal brain injury, tissue-specific immunity, intrauterine inflammation, neuroimmune activation, γ/δ T cell, IFNγ

## Abstract

Intrauterine inflammation impacts prenatal neurodevelopment and is linked to adverse neurobehavioral outcomes ranging from cerebral palsy to autism spectrum disorder. However, the mechanism by which a prenatal exposure to intrauterine inflammation contributes to life-long neurobehavioral consequences is unknown. To address this gap in knowledge, this study investigates how inflammation transverses across multiple anatomic compartments from the maternal reproductive tract to the fetal brain and what specific cell types in the fetal brain may cause long-term neuronal injury. Utilizing a well-established mouse model, we found that mid-gestation intrauterine inflammation resulted in a lasting neutrophil influx to the decidua in the absence of maternal systemic inflammation. Fetal immunologic changes were observed at 72-hours post-intrauterine inflammation, including elevated neutrophils and macrophages in the fetal liver, and increased granulocytes and activated microglia in the fetal brain. Through unbiased clustering, a population of Gr-1+ γ/δ T cells was identified as the earliest immune cell shift in the fetal brain of fetuses exposed to intrauterine inflammation and determined to be producing high levels of IFNγ when compared to γ/δ T cells in other compartments. In a case-control study of term infants, IFNγ was found to be elevated in the cord blood of term infants exposed to intrauterine inflammation compared to those without this exposure. Collectively, these data identify a novel cellular immune mechanism for fetal brain injury in the setting of intrauterine inflammation.

## Introduction

Maternal inflammation is associated with fetal brain injury and long-term neurodevelopmental impairment. Intrauterine immune activation – whether from maternal autoinflammatory disease, infection, or microbial dysbiosis– contributes to offspring neurologic diseases from schizophrenia to autism spectrum disorder (ASD) (1–5). Overt symptoms of intrauterine inflammation, or clinical chorioamnionitis, occur in a small number of deliveries and likely do not identify the majority of infants at risk from exposure to intrauterine inflammation (6). In contrast, histological chorioamnionitis (HCA), postpartum pathologic evidence of intrauterine inflammation, occurs in 10-20% of term deliveries with an increasing prevalence observed with decreasing gestational age (6–8). However, histologic examination of postpartum tissue cannot explain how inflammation at the maternal-fetal interface initiates long-lasting injury in an entirely distinct anatomic compartment, the fetal brain.

Immune activation in the central nervous system is posited to be a necessary mechanistic link between a prenatal immune insult and select neurological disorders (1). Patients with schizophrenia have more activated microglia as measured by positron emission tomography (PET) scan uptake and more dense microglia in post-mortem studies (9, 10). Patients with ASD have greater T cell infiltration at the blood brain barrier in post-mortem studies (11). These two distant immunologic findings – *in utero* and post-mortem – outline the wide knowledge gap in the actual pathogenesis of these disorders. More mechanistic evidence exists for immune cell development and the “immune education” that occurs prenatally, demonstrating that immunologic memory begins *in utero* and influences immune cell responsiveness to allergens, pathogens, and other antigens later in life (12, 13). Neuroimmune “education” may also occur prenatally; for example, one mouse model of ASD is initiated by injections of IL-17A to the fetal brain (14). However, this model does not address how subclinical intrauterine inflammation could be transmitted across the maternal-fetal dyad and induce fetal brain injury.

Our laboratory has established a mouse model of low-dose intrauterine inflammation (IUI) that leads to fetal neuronal damage, including abnormal neuronal morphology and decreased dendritic counts in cortical culture (15–17). In this model, exposure to IUI induces transcriptional and metabolic alterations to both fetal and neonatal brains, and a reduction in early postnatal neurogenesis (17–19). This model of IUI also causes postnatal white matter damage and behavior abnormalities in adult offspring (20). These findings demonstrate the utility of this model for studying IUI as a cause of fetal brain injury with long-term consequences. However, the mechanisms by which IUI translate to neuronal damage have not been fully explored.

Therefore, the objective of this study is to identify the cellular immunologic changes that propagate across anatomic compartments from the uterus to the fetal brain. Immune cell populations and their responses are tissue-specific and must be analyzed as such. Using an established mouse model of IUI, immune cell composition and function were investigated by flow cytometry and ELISA over multiple prenatal time points in both maternal and fetal murine tissues, and human cord blood. We hypothesize that IUI will induce a cascade of cellular immune alterations, ultimately causing abnormal immune cell trafficking and activation within the fetal brain itself.

## Materials and Methods

### Study Design

The goals of this study were to elucidate immune compositional and functional shifts associated with fetal brain injury in a mouse model and to correlate our findings to human cord blood samples. Flow cytometry and ELISA data are each compiled from a minimum of two experiments per time point with our mouse model and an N=4-6 mice/group/experiment. Human cord blood was obtained from an existing biorepository of cord blood serum collected for detection of biomarkers of fetal pathologies.

### Animal Model

These studies utilize a well-established mouse model of intrauterine inflammation created by our lab and resulting in fetal and postnatal brain injury (16, 17). For these experiments, timed-pregnant CD-1 mice were purchased from Charles River Laboratories (Wilmington, MA) and arrived at our animal facility at embryonic day 11 (E11). On E15, pregnant mice underwent a mini-laparotomy, while under isoflurane anesthesia. Each mouse received an injection into their right uterine horn, between the first and second amniotic sacs proximal to the cervix, as previously described (21–23). Each injection was either 100µl sterile phosphate buffered saline (PBS; saline control group) or 50µg/100µl lipopolysaccharide (LPS)/sterile PBS (IUI-exposed group). LPS was from *E. coli* 055:B5 (Sigma, St. Louis, MO). Mice were then euthanized by CO_2_ either 48 or 72 hours post-IUI, with tissues collected only from mice that were still pregnant. All animal care and use procedures are approved by the University of Pennsylvania IACUC.

### Tissue Collection

At either 48 or 72 hours post-IUI, mice were euthanized and tissues were collected into and washed with 10% charcoal-stripped fetal bovine serum (FBS; Gemini Bio, Sacramento, CA) in Hank’s balanced salt solution (HBSS) on ice. Maternal spleen was collected. Amniotic fluid was removed from each gestational sac with a 1ml syringe. The uterus was bisected and gestational sacs with fetal membranes and placentae were removed. The decidua was then scraped from uterus with a glass microscope slide into HBSS + 10% FBS on ice. Fetal tissues, placentae, fetal liver and fetal brains, were collected from the four fetuses proximal to the injection site and washed in HBSS + 10% FBS on ice. Tissues were either flash frozen in liquid nitrogen and stored at -80°C or immediately processed into a single cell suspension for flow cytometry.

### Single Cell Suspension

Preparation of tissues for flow cytometry follows a protocol established with the placenta (24). Placentae were minced into 2mm pieces, suspended in 5ml of digest solution (HBSS + 10% FBS + 1mg/ml Collagenase IV, Gibco, Gaithersburg, MD), and incubated in a 37°C water bath for 30 minutes. Placentae (post-digest treatment), maternal spleens, decidua, fetal livers, and fetal brains were mechanically pressed through a 70µm cell strainer. The strainer was rinsed with 10ml of HBSS and strained cells were passed through a 40µm cell strainer into a 50ml conical tube. Cells were centrifuged at 1500rpm, 4°C for 10 minutes. Red blood cells were eliminated from placentae, maternal spleens, decidua, and fetal livers by suspension in 3ml of ACK lysing buffer (Gibco, Gaithersburg, MD) and incubation at room temperature for 10 minutes. ACK-treated cells and amniotic fluid samples were rinsed in 10ml of HBSS and centrifuged at 1500rpm, 4°C for 5 minutes. Single cell suspensions from maternal spleen, decidua, placentae, fetal livers, and amniotic fluid were suspended in 1ml of FACS buffer (PBS + 2% FBS + 20µM EDTA) and transferred into 5ml round-bottom tubes through 35µm filter caps. Cells were stored on ice until ready for staining.

### Isolation of Mononuclear Cells From Fetal Brains

Fetal brain single cell suspension was suspended in 5ml 35% Percoll and was slowly layered on top of 5ml 70% Percoll in a 15ml conical tube. Cells in Percoll gradient were centrifuged at 1000xg, 4°C, for 20 minutes without centrifuge break. Myelin and glial cells were removed from the top of the gradient and then 1ml at the interface was collected, containing mononuclear cells. Mononuclear cells were washed in 10ml HBSS and centrifuged at 1500rpm, 4°C for 10 minutes. Cells were resuspended in 1ml FACS buffer and transferred into 5ml round-bottom tubes through 35µm filter caps.

### Cell Staining for Flow Cytometry

Cells were transferred to a 96-well round-bottom plate for staining, washed with 200µl of PBS, and the plate was centrifuged at 1500rpm, 4°C for 5 minutes. Cells were resuspended in 100µl of Live/Dead Fixable Aqua (Invitrogen, Waltham, MA) diluted 1:1000 in PBS and incubated for 30 minutes at 4°C in the dark. Cells were washed twice with 200µl of FACS buffer, centrifuging the plate between washes at 1500rpm, 4°C for 5 minutes. Cells were then resuspended in an extracellular antibody mix and incubated for 30 minutes at 4°C in the dark. Extracellular antibody mixes included: anti-CD3e-BV785, anti-CD4-BV605, anti-CD11c-PE.Cy7, anti-CD19-APC, anti-CD45-BV711, anti-CD49b-PacificBlue, anti-CD80-PacificBlue, anti-F4/80-FITC, anti-Ly6G-AF700, anti-TCRγ/δ-PerCP.Cy5.5 (BioLegend, San Diego, CA); anti-CD8a-APC.efluor780, and anti-Gr1-AF700 (eBioscience, San Diego, CA). The antibody clone and dilution used are listed in **Table 1**. Cells were washed twice with 200µl of FACS buffer, centrifuging between washes at 1500rpm, 4°C for 5 minutes. Cells were resuspended in 200µl fixation/permeabilization reagent (Transcription Factor Staining Buffer Set, eBioscience, San Diego, CA) and incubated for 30 minutes at 4°C in the dark. Cells were washed twice with 200µl of permeabilization buffer (Transcription Factor Staining Buffer Set, eBioscience, San Diego, CA), centrifuging at 2000rpm, 4°C for 5 minutes between washes. Cells were resuspended in anti-FoxP3-PE (eBioscience, San Diego, CA) diluted 1:300 in 100µl permeabilization buffer and incubated for 30 minutes at room temperature in the dark. Cells were washed twice with 200µl of permeabilization buffer, centrifuging at 2000rpm, 4°C for 5 minutes between washes. Cells were then resuspended in 200µl of FACS buffer and transferred into 5ml round-bottom tubes through 35µm filter caps and stored at 4°C in dark. Single-stained CompBeads (BD Biosciences, Franklin Lakes, NJ) were used as compensation controls to create a compensation matrix, and fluorescence minus one-stained splenocytes and placental cells (FMOs) were used as gating controls to draw gates. See gating scheme (**Figure S1**). All samples were analyzed by an LSR-II flow cytometer (BD Biosciences, Franklin Lakes, NJ).

**Table 1:** Flow Cytometry Antibodies.

Target	Clone	Fluor	Dilution	Catalog #	Brand
CD11c	N418	PE-Cy7	1:100	117318	BioLegend
CD19	6D5	APC	1:200	115512	BioLegend
CD3e	17A2	BV785	1:400	100232	BioLegend
CD4	RM4-5	BV605	1:200	100548	BioLegend
CD45	30-F11	BV711	1:200	103147	BioLegend
CD49b	DX5	Pacific Blue	1:100	108918	BioLegend
CD80	16-10A1	Pacific Blue	1:100	104724	BioLegend
CD8a	53-6.7	APC-ef780	1:200	47-0081-82	eBioscience
F4/80	BM8	FITC	1:100	123108	BioLegend
FoxP3	FJK-16s	PE	1:300	12-5773-82	eBioscience
Gr-1	RB6-8C5	AF700	1:200	56-5931-82	eBioscience
IFNg	XMG1.2	BV421	1:200	563376	BD Biosciences
IL-17A	TC11-18H10.1	PE	1:200	506904	BD Biosciences
Ly6G	1A8	AF700	1:200	127622	BioLegend
TCRb	H57-597	APC-Cy7	1:200	109220	BioLegend
TCRg/d	GL3	PerCP-Cy5.5	1:200	118118	BioLegend
TNFa	MP6-XT22	FITC	1:200	554418	BD Biosciences

### Cytokine ELISAs

Tissues to be used for ELISA were flash frozen in liquid nitrogen at the time of tissue harvest and were stored at -80°C. Amniotic fluid samples to be used for ELISA were centrifuged at 3000rpm for 5 minutes and the supernatant was flash frozen for future use. Protein extracts were made from placentae or fetal liver tissue by submerging 50mg of frozen tissue in 1ml of RIPA buffer with cOmplete mini protease inhibitor cocktail (Roche, Basel, Switzerland) in a 2ml round bottom tube with a 5mm steel bead. Tissue was homogenized on Tissue Lyser II (Qiagen, Venlo, Netherlands) for 10 minutes at 30/second. Homogenate was rested on ice for 40 minutes and then centrifuged at 14000xg for 10 minutes. Total protein in supernatant was quantified by the BCA protein assay kit (Pierce, Rockford, IL) following the manufacturer’s protocol. Quantikine ELISA kits (R&D Systems, Minneapolis, MN) for IL-6, IL-10, IFNγ, CCL3, and CCL5 were used to measure cytokine levels, following the manufacturer’s protocol. Cytokine levels were normalized to total protein.

### Intracellular Cytokine Staining

Fetal brains, decidua and maternal spleen were collected 72 hours post-intrauterine injection of LPS and processed into a single cell suspension as noted above. CD3+ cells were isolated from the single-cell suspension using mouse CD3e microbeads (Miltenyi Biotec, Bergisch Gladbach, Germany) following the manufacturer’s protocol. Isolated CD3+ cells were resuspended at 10^6^ cells/ml in T cell stimulation media: RPMI-1640 with L-glutamine (Corning Inc., Corning NY) supplemented with 50µM beta-mercaptoethanol (Sigma-Aldrich, St. Louis, MO), 1X non-essential amino acids, 1X sodium pyruvate, 1X HEPES buffer (Gibco, Gaithersburg, MD), 1X Cell Stimulation Cocktail, 1X Protein Transport Inhibitor Cocktail (eBioscience, San Diego, CA), and 10% FBS. 100µl of each sample was plated on a 96-well round-bottom plate and incubated for 4 hours at 37°C. Cells were centrifuged at 400xg, 4°C, for 5 minutes, washed with PBS, resuspended in Live/Dead Fixable Aqua 1:2000 in 100µl of PBS, and incubated for 30 minutes at 4°C. Cells were then washed twice with FACS buffer; stained with extracellular markers, anti-CD45-BV711, anti-CD3ε-BV785, anti-TCRγ/δ -PerCP-Cy5.5, anti-TCRβ-APC-Cy7 (BioLegend, San Diego, CA); and incubated for 30 minutes at 4°C. Cells were washed twice with FACS buffer and resuspended in Fixation/Permeabilization reagent (eBioscience, San Diego, CA) for 30 minutes at 4°C. Cells were washed twice with Permeabilization Buffer and then incubated for 30 minutes at 4°C with intracellular cytokine staining antibodies: anti-TNFα-FITC (BioLegend, Sand Diego, CA), anti-IFNγ-BV421, and anti-IL-17A-PE (BD Biosciences, San Jose, CA). Cells were washed twice with Permeabilization Buffer and then resuspended in FACS buffer and stored at 4°C in dark until run on flow cytometer.

### Flow Cytometry Analysis

Cells were analyzed on an LSR-II flow cytometer (BD Biosciences, San Jose, CA) running FACSDiva software (BD Biosciences, San Jose, CA) in the University of Pennsylvania Flow Core. Flow cytometry data was then analyzed using FlowJo (BD Life Sciences, Ashland, OR) to identify immune cell subsets. High-dimensional, unbiased cell clustering and visualization tools were used to further analyze flow cytometry data. Specifically, t-SNE and FlowSOM algorithms were downloaded from the FlowJo Exchange and implemented using R/Bioconductor (25).

### Case-Control Study

Cord blood was obtained from a previously published cohort of singleton pregnancies (N=723) (26, 27) of which 77 specimens were utilized in this study. Case specimens were selected as term neonates (>37 weeks gestational age at delivery) with documented HCA (N=41). Control specimens were also from term neonates (>37 weeks) that had placental histological examination but no HCA and were frequency matched for maternal race (N=36). All participants provided informed consent and the study was approved by the Institutional Review Board at the University of Pennsylvania (IRB #807678). Cord blood serum was analyzed by high sensitivity Milliplex assay (Millipore Sigma, Burlington, MA) for IFNγ and TNFα.

### Statistical Analysis

Statistical analyses were performed using GraphPad Prism. Data for every analysis is compiled from at least two experiments with 4-6 samples/group/experiment. Flow cytometry data was analyzed for differences between IUI and control samples collected at the same time – not for differences over time. All data was assessed for normality by Shapiro-Wilk test. Non-normally distributed data was analyzed by Mann-Whitney test. Normally distributed data was analyzed by unpaired student’s t-test, with Welch’s correction applied if the two groups differed in variance by F test. ELISA data and intracellular cytokine staining were analyzed by two-way ANOVA, followed by Tukey’s multiple comparison test if initial ANOVA was significant. Human cord blood data was non-normally distributed by Shapiro-Wilk test and analyzed for differences by Mann-Whitney test. Cord blood data was also divided by fetal sex and analyzed by two-way ANOVA to determine if fetal sex was a significant factor contributing to variance.

## Results

### Intrauterine Inflammation Alters Local, but Not Systemic, Immune Populations

Our previous work utilizing our model of intrauterine inflammation (IUI) has demonstrated minimal maternal immune activation after IUI, specifically noted by a lack of maternal serum cytokine elevation (17). To confirm the hypothesis that the maternal systemic immune system is not impacted by IUI, we compared maternal splenic immune populations by flow cytometry from mice with IUI compared to saline-treated controls. From each spleen sample, 10^6^ events were run on the flow cytometer, leading to the requisition of approximately 5x10^5^ CD45+ cells (**Figure S2A**). At both 48- and 72-hours post-uterine injection, none of the following immune populations examined were significantly different between the two groups: macrophages, CD4+ T cells, CD8+ T cells, regulatory T cells, γ/δ T cells, B cells, NK cells, neutrophils, and dendritic cells (**Figure S2B–J**).

**Figure 1:**
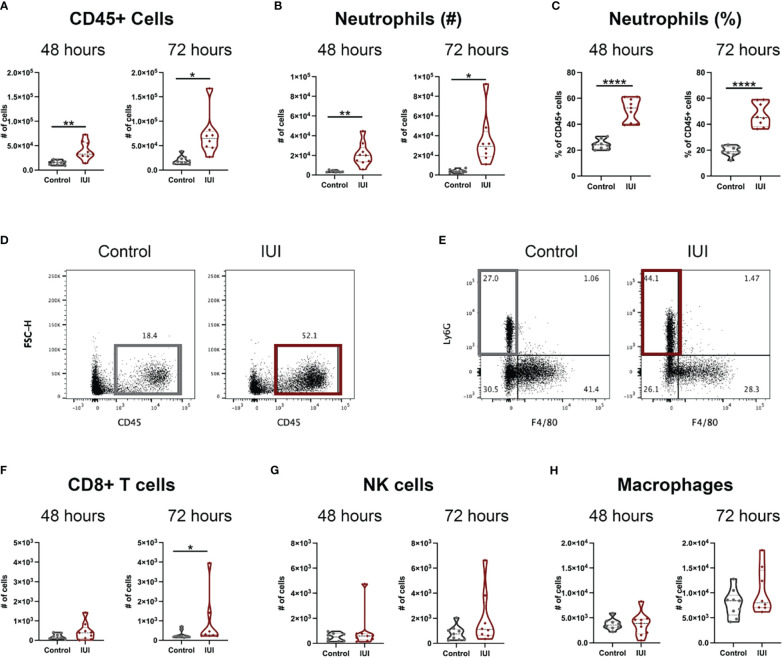
Intrauterine inflammation causes sustained influx of neutrophils to the decidua. Decidua was removed from the uterus at 48 and 72 hours post-uterine injection. All decidual cells were analyzed by flow cytometry to have full counts of **(A)** CD45+ cells. **(D)** Representative flow plots of total CD45+ cells are gated on non-debris, singlet, live cells. Immune cell subsets were reported both as cell counts and as a percent of total CD45+ cells, including **(B, C)** neutrophils, **(F)** CD8+ T cells, **(G)** NK cells, and **(H)** macrophages. **(E)** Representative flow plots of neutrophils were gated on non-debris, singlet, live, CD45+, CD19-, CD4-, CD8- cells. Significance was determined by unpaired t-test with Welch’s correction if positive F-test. *p<0.05, **p<0.01, ****p<0.0001.

Decidua, the specialized uterine lining that develops during pregnancy, was collected from these same mice to examine immune changes local to the uterus at 48- and 72-hours post-uterine injection. The presence of IUI increased numbers of CD45+ cells in decidual tissue (**Figures 1A, D**), primarily due to an influx of decidual neutrophils (**Figures 1B, C, E**). This neutrophil influx was evident as both an increase in absolute number of decidual neutrophils (**Figures 1B**) and an increase in neutrophil frequency of decidual leukocytes (**Figure 1C**). The decidual neutrophil increase persisted in the mice with IUI at 72 hours post-uterine injection (**Figures 1B, C**) and these mice also had a slight elevation in decidual CD8+ T cells at 72 hours post-uterine injection (**Figures 1F**). The two most prevalent decidual immune cells, NK cells and macrophages (28, 29) did not differ between the IUI and control groups (**Figures 1G, H**).

### Placentae and Amniotic Fluid Have Elevated Leukocyte Counts and Chemokine Levels Following Intrauterine Inflammation

**Figure 2:**
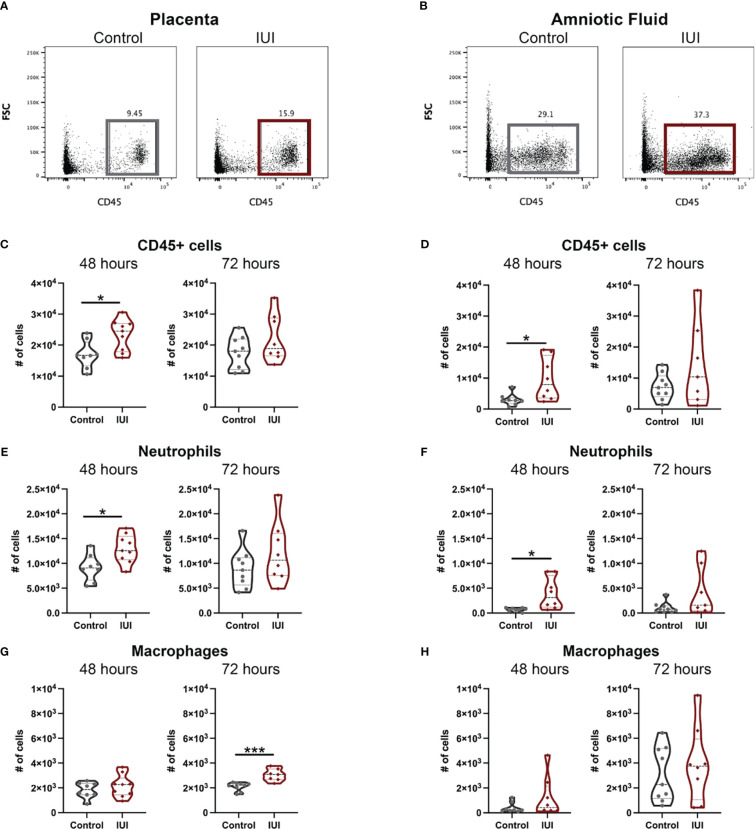
Intrauterine inflammation increases leukocytes found in the placenta and amniotic fluid. Placentae adjacent to the injection site and total amniotic fluid were collected at 48 and 72 hours post-uterine injection and immune cells were analyzed by flow cytometry. Representative flow plots of CD45+ cells in the **(A)** placenta and **(B)** amniotic fluid were gated on non-debris, singlet, live cells. **(C, D)** CD45+ cells, **(E, F)** neutrophils, and **(G, H)** macrophages were counted at each time point. Cell counts were tested for normal distribution by Shapiro-Wilk test. Normally distributed data was then analyzed by unpaired t-test and non-normally distributed data was analyzed by Mann-Whitney test. *p<0.05, ***p<0. 001.

To test the hypothesis that IUI would alter immune responses both within and across the placenta, flow cytometry was used to identify immune cells in the placentae and amniotic fluid of mice with IUI and controls. At 48 hours post-uterine injection, both the placentae and amniotic fluid of mice exposed to IUI had more total leukocytes than controls, but these levels normalized by 72 hours post-exposure (**Figures 2A–D**). Neutrophil increases in both the placenta and amniotic fluid accounted for most of the leukocyte increase in mice with IUI at 48 hours post-uterine injection and neutrophil counts in the placentae and amniotic fluid of mice with IUI also had normalized by 72 hours post-uterine injection (**Figures 2E, F**). Placentae, but not amniotic fluid, from mice with IUI had a small increase in macrophages (**Figures 2G, H**), but no other immune cell differences from control animals were observed by 72 hours post-uterine injection.

**Figure 3:**
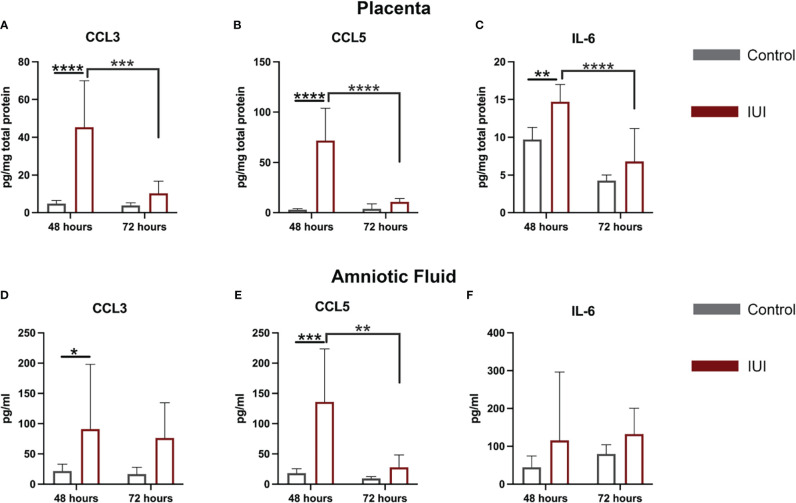
Elevated chemokines found in the placenta and amniotic fluid following intrauterine inflammation. Cytokines were measured in the placenta and amniotic fluid by ELISA, from samples collected at 48 and 72 hours post-uterine injection. **(A–C)** Placental ELISA values were normalized to total placental protein measured by BCA, while **(D–F)** amniotic fluid ELISA values were normalized to fluid volume. Significant differences were determined by two-way ANOVA followed by *post-hoc* Tukey’s multiple comparison test. *p<0.05, **p<0.01, ***p<0.001, ****p<0.0001.

Given the increase in CD45+ cells in the placentae and amniotic fluid of mice with IUI, we asked whether this was accompanied by an increase in chemokines that may recruit CD45+ cells to these tissues. Our previous work with this model of IUI had demonstrated significant elevations in CCL3, CCL5, and IL-6 in both the placenta and amniotic fluid at just 6 hours post-exposure (16, 17, 30), so we investigated whether these chemokines remained elevated at later time points. CCL5 was the most increased: 31-fold in the placenta and 8-fold in the amniotic fluid of mice with IUI at 48 hours post-uterine injection; but protein levels of this chemokine normalized to control levels by 72 hours post-uterine injection (**Figures 3B, E**). CCL3 was also elevated in the placenta and amniotic fluid in mice with IUI at 48 hours and normalized by 72 hours post-uterine injection (**Figures 3A, D**). IL-6 was elevated in the placenta but not the amniotic fluid of mice with IUI at 48 hours and was not significantly increased in either tissue at 72-hours post-injection (**Figures 3C, F**).

### Exposure to Intrauterine Inflammation Alters Immune Populations in the Fetal Liver

**Figure 4:**
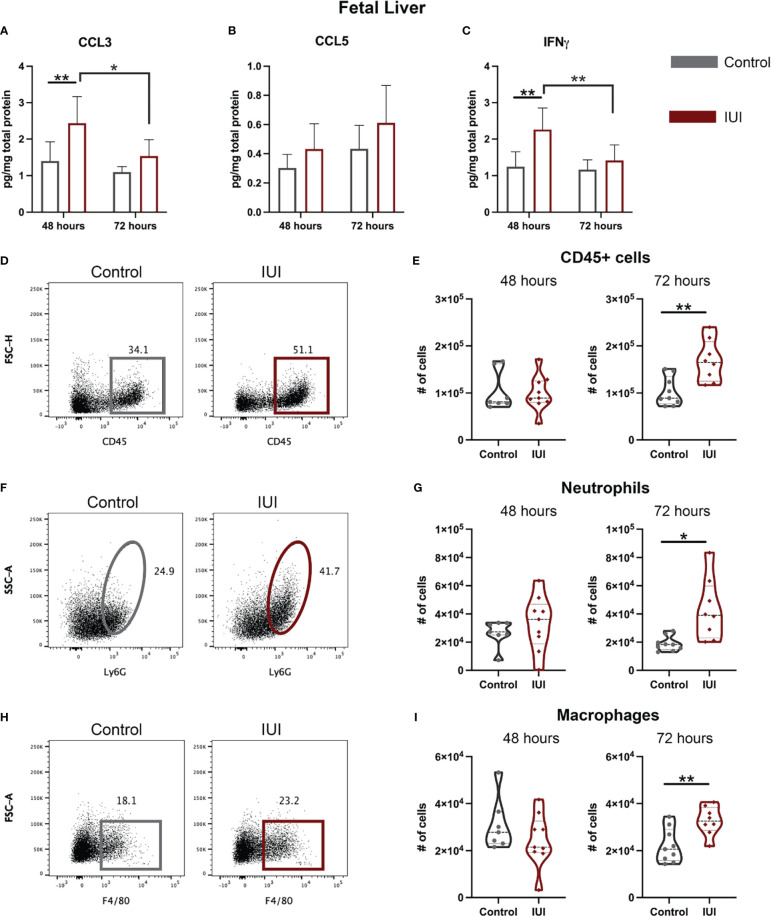
Neutrophil and cytokine elevation in the fetal liver demonstrate systemic fetal inflammation. Fetal livers were collected at 48 and 72 hours post-uterine injection and either frozen or processed for flow cytometry. **(A–C)** Frozen fetal livers were tested for cytokines by ELISA and normalized to total protein. Fresh fetal livers were analyzed by flow cytometry for immune cells. **(D, E)** Total CD45+ cells, **(F, G)** neutrophil, and **(H, I)** macrophage cell counts are shown. **(D)** Representative flow plots of CD45+ cells were gated on non-debris, singlet, live cells. **(F)** Representative flow plots of neutrophils were gated on non-debris, singlet, live, CD45+, CD3-, CD19- cells. **(H)** Representative flow plots of macrophages were gated on non-debris, singlet, live, CD45+, CD3-, CD19- cells. Cell count data was analyzed by Mann-Whitney test. Cytokine data were analyzed by two-way ANOVA with *post-hoc* Tukey’s multiple comparison test. *p<0.05, **p<0.01.

Given that immune alterations were detected in the amniotic fluid of IUI-exposed mice, we hypothesized that the fetal immune system would be perturbed. In the fetal livers of mice exposed to IUI, CCL3 and IFNγ were elevated at 48 hours post-uterine injection and had normalized by 72 hours (**Figures 4A, C**). There was no significant difference in CCL5 levels between IUI-exposed and control fetuses (**Figure 4B**). No cellular immune changes were noted at 48 hours in the fetal liver. However, following the observed cytokine elevation at 48 hours, at 72 hours post-uterine injection, fetuses exposed to IUI had more CD45+ cells (**Figures 4D, E**), neutrophils (**Figures 4F, G**), and macrophages (**Figures 4H, I**) than control fetuses in their fetal livers.

### Activated Microglia and Granulocytes Are Increased in Fetal Brains From in Fetuses Exposed to Intrauterine Inflammation

**Figure 5:**
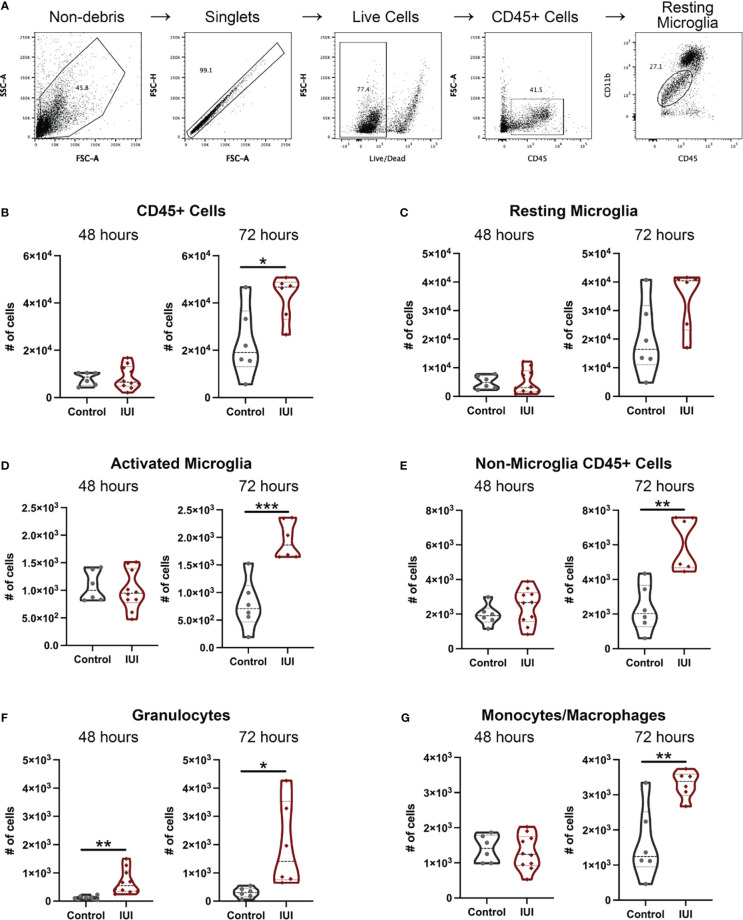
Intrauterine inflammation results in fetal microglial activation and an increase in non-microglial immune cells in the fetal brain. Fetal brains were collected at 48 and 72 hours post-uterine injection and processed for flow cytometry. **(A)** The gating scheme used to identify immune cells in the fetal brain is shown. **(B)** Total CD45+ cells and immune cell subtypes were counted. Subtypes include: **(C)** resting microglia, **(D)** activated microglia, **(E)** all non-microglia CD45+ cells, **(F)** granulocytes, and **(G)** monocytes/macrophages. All cell count data was analyzed by Shapiro-Wilk test to establish data distribution. Normally distributed data were analyzed by unpaired t-test and non-normally distributed data were analyzed by Mann-Whitney test. *p<0.05, **p<0.01, ***p<0.001.

To address whether an inflammatory insult in the uterus could ultimately alter immune cell composition and function in the fetal brain, we isolated mononuclear cells from fetal brains and identified the immune cell types by flow cytometry (gating scheme in **Figure 5A**). At 48 hours post-uterine injection, the only detectable difference in the fetal brain immune cells was a 5-fold increase in granulocytes in the IUI-exposed group (**Figure 5F**). However, by 72 hours post-uterine injection, fetal brains from IUI-exposed dams had more overall CD45+ cells (**Figure 5B**) and specifically non-microglial CD45+ cells (**Figure 5E**), including 7-fold more granulocytes (**Figure 5F**). IUI-exposure increased the numbers of activated microglia and macrophages but did not affect the numbers of resting microglia (**Figures 5C, D, G**).

### Unbiased Analyses Identify Gr-1+ γ/δ T Cells as Highly Increased in the IUI-Exposed Fetal Brains

**Figure 6:**
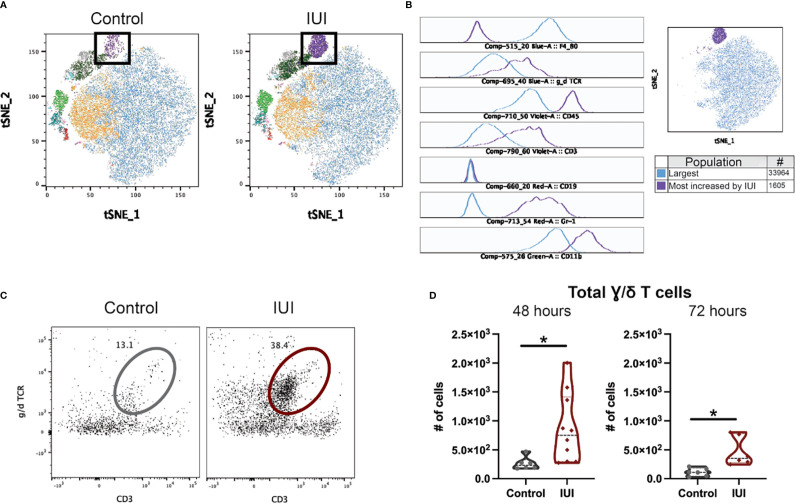
Intrauterine inflammation increases γ/δ T cells in the fetal brain. Fetal brains were collected at 48 and 72 hours post-uterine injection and processed for flow cytometry. CD45+ cells in the fetal brain at 72 hours were analyzed by FlowJo plug-ins tSNE and flowSOM to visualize and identify clusters, respectively. **(A)** Fetal brain CD45+ cells from saline-control and IUI-exposed dams were visualized by tSNE and colored by flowSOM populations. Black box indicates the most increased population by IUI. **(B)** Histograms of marker expression for two populations identified by flowSOM: the largest population (blue) and the population most increased by IUI-exposure (purple). **(C)** Representative flow plots of the γ/δ T cells in the fetal brain, gated on non-debris, singlet, live, CD45+, non-microglia, CD19- cells. **(D)** γ/δ T cells were counted in each fetal brain sample. All cell count data was analyzed by Shapiro-Wilk test to establish data distribution. Normally distributed data were analyzed by unpaired t-test and non-normally distributed data were analyzed by Mann-Whitney test. Normally distributed data with positive F-test for unequal variance were analyzed by unpaired t-test with Welch’s correction. *p<0.05.

Given limited studies on what immune cells can be found in the fetal brain, we relied on unbiased clustering algorithms to determine the immune population with the greatest difference between fetal brains from IUI-exposed and control dams. tSNE analysis creates a visual map of the different CD45+ cells found in the fetal brain, with a clear population enriched in the brains of fetuses exposed to IUI (black box, **Figure 6A**). The flowSOM algorithm generated ten different clusters of immune cells in the fetal brain (different colored clusters in **Figure 6A**). The absolute difference between the frequency of each flowSOM cluster in the fetal brains of IUI-exposed *versus* control mice was calculated and the population with the greatest difference was identified (purple population, **Figures 6A, B**). This population accounted for 7% of all CD45+ cells in the brains from IUI-exposed fetuses, but less than 1% of CD45+ cells in brains of control fetuses. This immune cell population was Gr-1+ CD11b+ γ/δ T cells (histograms in **Figure 6B**). Total γ/δ T cells were elevated in the fetal brains from IUI-exposed dams at both 48 and 72 hours post-uterine injection (**Figures 6C, D**). Total γ/δ T cells were also increased in the decidua of IUI-exposed dams at 72 hours post-uterine injection (**Figure S3A**), but were not changed in the spleen (**Figure S2F**), placenta, amniotic fluid or fetal liver (**Figures S3B–D**) by IUI.

### γ/δ T Cells in the Fetal Brain, but Not the Decidua or Maternal Spleen, Produce IFNγ Following Intrauterine Inflammation

**Figure 7:**
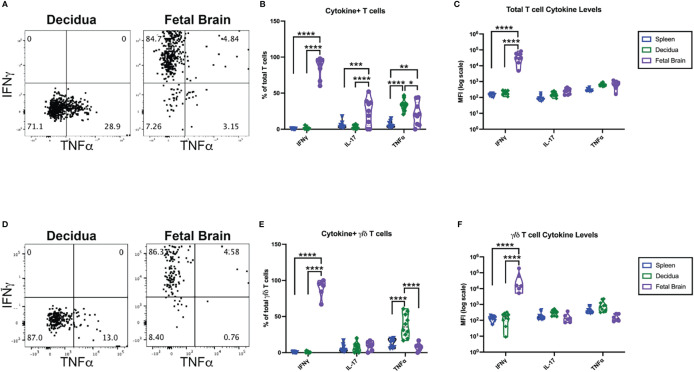
γ/δ T cells from the fetal brain, but not from the maternal spleen or decidua, are producing IFNγ;. CD3+ cells were isolated from tissues collected at 72 hours post-exposure to IUI. CD3+ cells were stimulated *in vitro* and stained for intracellular cytokines as well as T cell receptor subtype. **(A)** Representative flow plots from the decidua and fetal brain are gated on non-debris, singlet, live, CD45+, CD3+ cells. **(D)** Representative flow plots of cytokines in γ/δ T cells followed the same gating scheme, with the additional gates of TCRβ- and TCRγδ+. Cytokine expression was analyzed as **(B)** the % of T cells or **(E)** % of γ/δ T cells that were positive for each cytokine and as the MFI of either **(C)** total T cells or **(F)** γ/δ T cells. Cytokine data was analyzed by two-way ANOVA with post-hoc Tukey’s multiple comparison test comparing the expression of a single cytokine between tissues. *p<0.05, **p<0.01, ***p<0.001, ****p<0.0001.

To elucidate the function of the γ/δ T cells in the IUI-exposed fetal brains, T cells were isolated from pregnant mice 72-hours after exposure to IUI from the maternal spleen, decidua, and fetal brain. T cells were stimulated *in vitro* for four hours and stained for intracellular cytokines. Cells were also stained for TCRβ and TCRγδ to differentiate between α/β and γ/δ T cells. Cytokine production by total T cells (**Figures 7A–C**) and specifically γ/δ T cells (**Figures 7D–F**) varied immensely by anatomic location. Approximately 10% of all splenic T cells produced at least one measurable cytokines, 33.7% of decidual T cells produced TNFα, and nearly 100% of T cells in the fetal brain produced at least one cytokine (**Figures 7A, B**). Of fetal brain γ/δ T cells, 9.1% produced IL-17A and 7.9% produced TNFα; while 89.4% of fetal brain γ/δ T cells produced IFNγ, with some γ/δ T cells producing multiple cytokines (**Figures 7D, E**). Whether examining total T cells or only γ/δ T cells, only those isolated from the fetal brain were making significant IFNγ (**Figures 7C, F**).

### IFNγ Is Elevated in Umbilical Cord Blood From Term Neonates With Histological Chorioamnionitis

**Figure 8:**
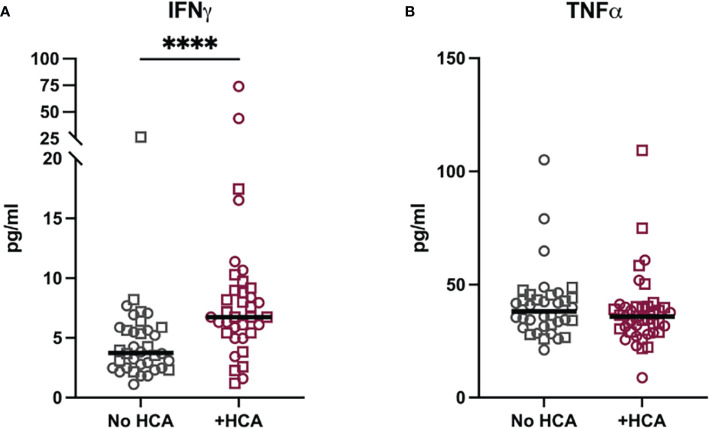
Cord blood from neonates with histological chorioamnionitis have elevated IFNγ. Cytokine levels were measured in a matched case-control study of cord blood plasma from term neonates (>37 weeks gestational age at delivery) with and without histological chorioamnionitis (HCA). **(A)** IFNγ and **(B)** TNFα were measured by Milliplex assay in 41 cases with HCA and 36 controls. Neonatal sex is noted by symbol shape: squares are male and circles are female. Sex did not impact variance in cytokine levels as measured by two-way ANOVA. Differences in cytokine levels were analyzed by Mann-Whitney test. ****p<0.0001.

Cord blood was collected from term pregnancies (delivery at greater than 37 weeks) and placentae from these pregnancies were examined for histological chorioamnionitis (HCA) as part of clinical care. Term HCA was used as a human correlate to our mouse model of low dose intrauterine inflammation without preterm birth, as HCA is defined by placental neutrophil infiltrates (7). A case control study was performed. Cases were defined by the finding of HCA on placental pathology (N=36) compared to controls who did not have HCA (N=41). Cases and controls were frequency matched by self-reported race (**Table 2**). IFNγ and TNFα were measured in cord blood by a high-sensitivity Luminex assay. IFNγ levels were 1.8-fold higher in cord blood from neonates with HCA compared to controls (p<0.0001), but TNFα was equivalent between cases and controls (**Figures 8A, B**), indicating an IFNγ-specific inflammatory signature in neonates with HCA. Fetal sex did not contribute to cytokine level variance.

**Table 2:** Demographic table of study participants.

	No HCA(N=36)	HCA (N=41)	p-Value
**Race**			
Black	29 (80.6%)	34.(82.9%)	1
Non-Black	7 (19.4%)	7 (17.1%)	
**Maternal Age (years)**			
Mean (SD)	25.9 (6.43)	23.2 (5.95)	0.06
**Birth Weight (g)**			
Mean (SD)	2980 (497)	3270 (627)	0.025
**5 min Apgar**			
<8	5 (13.9%)	3 (7.3%)	0.57
8+	31 (86.1%)	38 (92.7%)	

## Discussion

The developing brain is exquisitely sensitive to intrauterine inflammation (IUI) such that the inflammatory insult may be asymptomatic in the mother but may cause long-lasting neuronal damage in the fetus. This study identifies a cascade of immunological events across the maternal-fetal dyad, demonstrating an active local immune response in the absence of systemic inflammation. The immune response in each compartment differed in cellular content, cytokine activation, and reaction time, emphasizing the importance of tissue-specific immunity and validating the necessity to examine each compartment across the maternal-fetal dyad. Activated IFNγ+ Gr-1+ γ/δ T cells in the fetal brain are a novel population that may be essential for fetal brain injury and IFNγ-driven inflammation may be critical for both identifying neonates at risk of brain injury and understanding the specific pathogenesis of neuroimmune diseases from exposure to prenatal inflammation.

The mouse model used in this study recapitulates the clinical phenotype as exposed dams exhibit no sign of systemic illness or immunologic shifts. Yet, even in the absence of systemic immunological shifts, significant immune responses occur at the maternal-fetal interface and within the fetus. This model greatly differs from other models of maternal inflammation inducing fetal brain damage, in which polyI:C (3, 14, 31), LPS (32), IL-1β (33, 34), or pathogen-specific antigens (35) are injected systemically into the mother to mimic a viral-like illness (36, 37). Overt maternal illness occurs in only a minority of cases of IUI as indicated by histological chorioamnionitis (7, 8); as such, the model utilized in these studies more accurately recapitulates mechanism of disease in human pregnancy. Most cases of human IUI are thought to be caused by ascension of vaginal bacteria to the uterus (7). Maternal intestinal microbiota also influences the development of ASD in offspring, whereby the microbiota affects maternal immune and metabolic factors that reach the fetus and change fetal neurodevelopment (3, 5, 38, 39). Our model better mimics the subtle local inflammatory changes initiated by microbial products or microbial ascension to the uterus when compared to systemic inflammatory models.

Despite the different modes of inducing inflammation, our model demonstrates a neutrophil influx to the decidua, placenta, and amniotic fluid – which is consistent with other animal and human studies (37, 40–43). Neutrophil infiltrates to the placenta are also diagnostic of human histological chorioamnionitis (7). The local immune response at the maternal-fetal interface therefore appears similar whether the inflammatory insult is local or systemic. Importantly, this study demonstrates that a lack of maternal systemic immune activity does not reflect the immune activity of the maternal-fetal interface.

**Figure 9:**
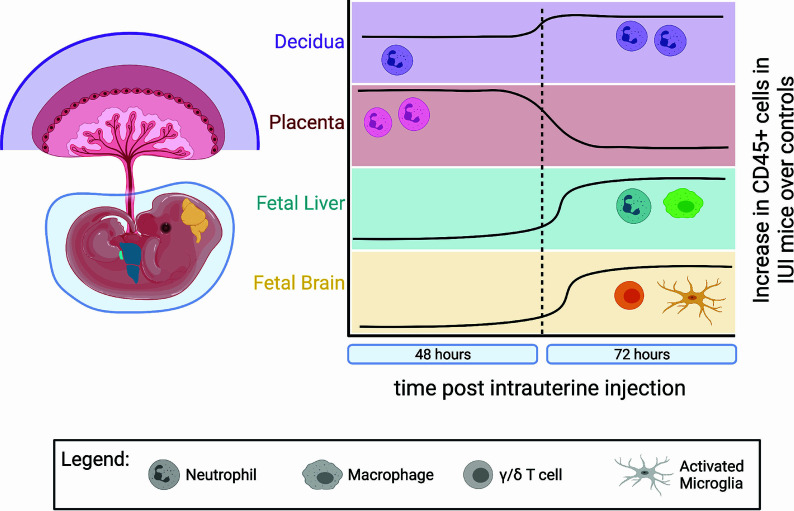
Temporal dynamics of immune infiltrates differed in each anatomic compartment. IUI-exposed mice had more CD45+ cells compared to controls in each examined tissue, but at different times post-uterine injection and with different immune subtypes. This figure demonstrates: the lasting neutrophil infiltrate in the decidua (purple) at 48 and 72 hours post-uterine injection; the neutrophil infiltrate to the placenta (pink) at 48 hours that normalizes to control levels by 72 hours; the increase in macrophages and neutrophils to the fetal liver (green) at 72 hours; and the increase in γ/δ T cells and activated microglia in the fetal brain (yellow) at 72 hours post-uterine injection. (Figure made using BioRender software.).

The duration of our study gives more context to the local immune cell infiltrates associated with IUI (**Figure 9**). Neutrophils are increased in the placenta and amniotic fluid at 48 hours post-uterine injection but have returned to control numbers by 72 hours. We find a similar trend with inflammatory cytokines in the placenta and amniotic fluid. Previous data from our laboratory has shown massive increases in IL-6 in the placenta and amniotic fluid in IUI-exposed pregnant mice at 6 hours post-uterine injection (17, 44). These current data show increases in CCL3, CCL5, and IL-6 in IUI-exposed pregnant mice at 48 hours, but by 72 hours post-uterine injection, these cytokines are returning to control levels. These data demonstrate a wave of inflammation through the placenta and amniotic fluid that is subsiding by 72-hours after initial insult.

**Figure 10:**
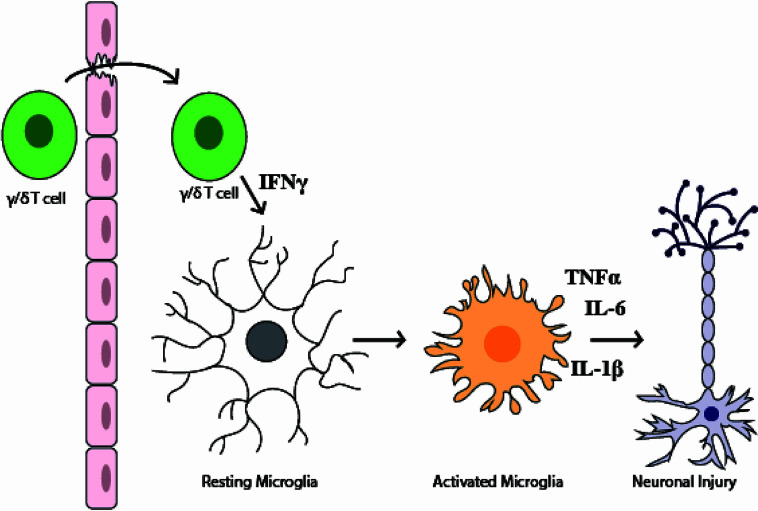
Model figure of the mechanism of fetal brain injury in the setting of intrauterine inflammation. This model demonstrates how γ/δ T cells in the fetal brain may lead to fetal brain injury. This model reflects both data in this paper and data from previously published literature that demonstrate that (1) the fetal blood brain barrier is weakened by maternal inflammation (2), during systemic inflammation γ/δ T cells may traffic to the brain (3), an increase in IFNγ+ γ/δ T cells are present in the fetal brain following IUI (4), IFNγ leads to microglial activation, and (5) prenatal microglial activation can cause neuronal injury.

Even as inflammation in the placenta and amniotic fluid is lessening by 72-hours post-uterine injection, cellular changes to the fetal immune system in IUI-exposed fetuses are just becoming apparent (**Figure 9**). The increase in macrophages and neutrophils in the fetal livers of IUI-exposed fetuses is a novel finding that indicates IUI not only may affect neurodevelopment but also immune system development. These findings are consistent with the immune irregularities and increased autoimmunity that are present in patients with ASD (1, 45). Fetal immune activation in our mouse model may be a direct response to LPS, as LPS is able to cross the placenta into fetal tissue and fetal TLR4 activation is necessary for LPS-induced brain damage (30, 44). Prenatal exposures to microbial products impact allergic sensitivity and tolerance induction of fetal immune cells of both mice and humans (46, 47). Fetal blood brain barrier permeability is influenced by maternal microbiota and immune activity (31, 48). Fetal inflammation following maternal immune activation leads to an accumulation of immune cells at the embryonic choroid plexus and a weakening of tight junctions at the blood brain barrier (31). This weakened barrier likely allows for the increased infiltration of non-microglial CD45+ cells that we find in the brains of IUI-exposed fetuses (**Figure 5E**) and provides a model for our findings (**Figure 10**).

The detection of increased IFNγ in mouse fetal livers of IUI-exposed fetuses and in human cord blood supports a more systemic immune activation occurring in the fetus in response to IUI. This systemic fetal inflammation may specifically initiate γ/δ T cell trafficking to the brain, as has been reported to occur neonatal sepsis (49). Similarly, in experimental autoimmune encephalomyelitis, γ/δ T cells are thought to be primed in the periphery and then travel to the central nervous system (CNS), where they are able to augment CNS pathology (50). Consistent with reports that demonstrate peripheral γ/δ T cells traffic to the brain (49–51), in the fetus γ/δ T cells are found in the skin, intestines and meninges (52). Recent work has demonstrated that the presence of meningeal γ/δ T cells in neonates are critical for short-term memory and synaptic plasticity (53). Interestingly, these physiologic γ/δ T cells produce IL-17 (53), while the γ/δ T cells that we detected in the fetal brain produce IFNγ, suggesting a different, possibly pathologic, activation state.

As the bidirectional crossing of immune cells between mother and fetus remains a poorly studied area (54), one might argue that the γ/δ T cells may be maternal in origin. Based on prior data showing very few maternal-derived cells in the fetal liver (24), we suggest that the fetal brain γ/δ T cells are likely fetal in origin. Furthermore, the earliest fetal γ/δ T cell progenitors to exit the thymus at E13 express IFNγ (52), which is consistent with the phenotype of the γ/δ T cells in this study.

The expression of both Gr-1 and CD11b on the γ/δ T cells in this study is consistent with their IFNγ production. Gr-1 has been used as a marker of IFNγ-producing rather than IL-17-producing γ/δ T cells in the setting of bacterial pneumonia (55), and CD11b+ γ/δ T cells are reported to produce IFNγ and to present exogenous antigens (56). Together the phenotype of these γ/δ T cells suggests they are responding to and exacerbating fetal inflammation in this model. The production of IFNγ by γ/δ T cells in the fetal brain may is one plausible mechanism by which these γ/δ T cells cause brain injury, as microglial exposure to IFNγ initiates a microglial activation state that is pro-inflammatory and neurotoxic (57, 58). Microglia impacted by inflammation in the “pre-microglial” phase from E14 to one month of life are particularly sensitive to inflammation and rearrange their chromatin landscape to create lasting changes in gene expression patterns (59, 60). These early epigenetic changes to microglia may explain the long-term neurobehavioral outcomes from prenatal exposure to inflammation.

While the initial injury may be prenatal, neuroimmune pathologies may be treated postnatally. In multiple animal models of prenatal neuronal injury and inflammation, postnatal treatments have reversed neurologic, immunologic, and even behavior abnormalities in the exposed offspring (35, 61–63). The potential for treatment heightens the necessity for early diagnosis. Given the absence of a systemic maternal signal for the presence of intrauterine inflammation, other avenues to identify fetuses and neonates at risk is mandated. Assessing the immunological state of a neonate is feasible with testing of the cord blood immediately following delivery. Our finding of increased IFNγ in human cord blood from neonates with HCA demonstrates the plausibility of cord blood containing an immune signature of neuronal injury. The increased cord blood IFNγ indicates that inflammation from HCA is not isolated to the placenta and impacts the fetus itself. Additionally, the elevation of IFNγ but not TNFα suggests an adaptive over innate fetal response to HCA.

One limitation to this study is that T cell development in the mouse occurs later in gestation than in humans. In mice, γ/δ T cells do not develop until E13 and α/β T cells mostly mature postnatally, whereas human T cell development begins at week 11-12 of pregnancy (64, 65). Therefore, the fetal immune response observed in the mouse may not be wholly reflective of that in a human fetus. However, postmortem studies from human preterm infants have shown γ/δ T cells in neonatal brains with injury (specifically periventricular leukomalacia) but not in preterm infants without brain injury (66), indicating that γ/δ T cells may be critical to fetal brain injury regardless of the presence of α/β T cells. γ/δ T cells were also found in a sheep model of fetal asphyxia-induced brain injury (66), suggesting a conserved role for γ/δ T cell in prenatal neuropathology, regardless of the initial insult. In addition, in multiple mouse models of neonatal brain injury (sepsis, hypoxia-ischemia), γ/δ T cells were required to cause brain injury, and depletion of γ/δ T cells was protective (49, 66). γ/δ T cells are also implicated in multiple ‘adult’ neuroinflammatory diseases including encephalitis, multiple sclerosis, and ischemic stroke (50, 51, 67, 68). These findings suggest a specific role for γ/δ T cells, rather than α/β T cells, in neuroimmune disease.

Overall, our data support a model of fetal brain injury where local intrauterine inflammation progresses to systemic fetal inflammation, which then creates the conditions for activated γ/δ T cells to cross the blood brain barrier, make IFNγ, and activate fetal microglia (**Figure 10**). The lack of maternal systemic inflammation posits a challenge for prenatal diagnosis of neurodevelopmental disorders. However, detection of IFNγ in human cord blood provides the potential for early neonatal diagnosis, and thus treatment, of prenatal neuronal damage. These findings demonstrate a complex immune response in the maternal-fetal dyad that participate in fetal brain injury from exposure to prenatal inflammation. Importantly, this work identifies a potential target and/or marker of fetal brain inflammation through the identification of γ/δ T cells in our mouse model and elevated IFNγ in term HCA. Being able to identify, through cord blood biomarkers, neonates at greatest risk for neuroimmune activation, may provide a novel opportunity for pharmacologic interventions to prevent adverse neurobehavioral outcomes associated with prenatal inflammation.

## Data Availability Statement

The original contributions presented in the study are included in the article/**Supplementary Material**. Further inquiries can be directed to the corresponding authors.

## Ethics Statement

The studies involving human participants were reviewed and approved by Institutional Review Board, University of Pennsylvania (IRB #807678). Written informed consent from the participants’ legal guardian/next of kin was not required to participate in this study in accordance with the national legislation and the institutional requirements. The animal study was reviewed and approved by Institutional Animal Care & Use Committee, University of Pennsylvania.

## Author Contributions

EL and ME conceptualized this study. EL, NT, LA, and AB determined the methodology and conducted the experiments. EL and PP analyzed the data. ME secured funding for this study. EL wrote the initial draft of this manuscript with significant review and editing by ME and LA. All authors contributed to the article and approved the submitted version.

## Funding

Eunice Kennedy Shriver National Institute of Child Health and Human Development (R01-HD076032) and March of Dimes (21-FY08-539 – Elovitz).

## Conflict of Interest

The authors declare that the research was conducted in the absence of any commercial or financial relationships that could be construed as a potential conflict of interest.

## Publisher’s Note

All claims expressed in this article are solely those of the authors and do not necessarily represent those of their affiliated organizations, or those of the publisher, the editors and the reviewers. Any product that may be evaluated in this article, or claim that may be made by its manufacturer, is not guaranteed or endorsed by the publisher.
