# Supplementary material for: IFNγ-Producing γ/δ T Cells Accumulate in the Fetal Brain Following Intrauterine Inflammation
Source: Front Immunol. 2021 Oct 4;12:741518. doi: 10.3389/fimmu.2021.741518 (PMC8524441; doi:10.3389/fimmu.2021.741518)
Supplement: Supplementary file 1 [file DataSheet_1.pdf]

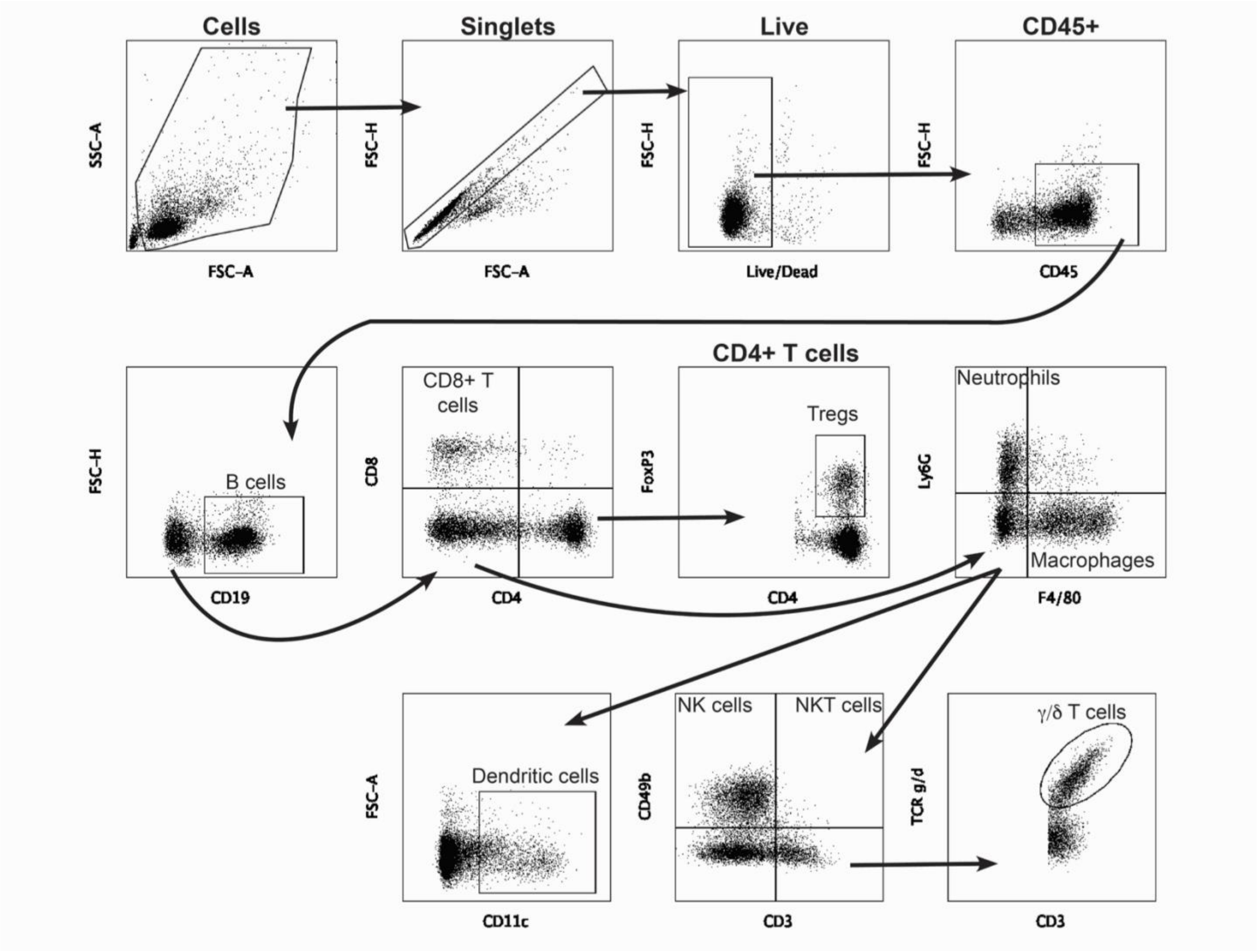

Figure S1: Flow Cytometry Gating Scheme. Cells from maternal spleen, decidua, placenta, amniotic fluid, and fetal liver were analyzed using the following gating scheme. Gates were determined with the use of fluorescence-minus-one controls.

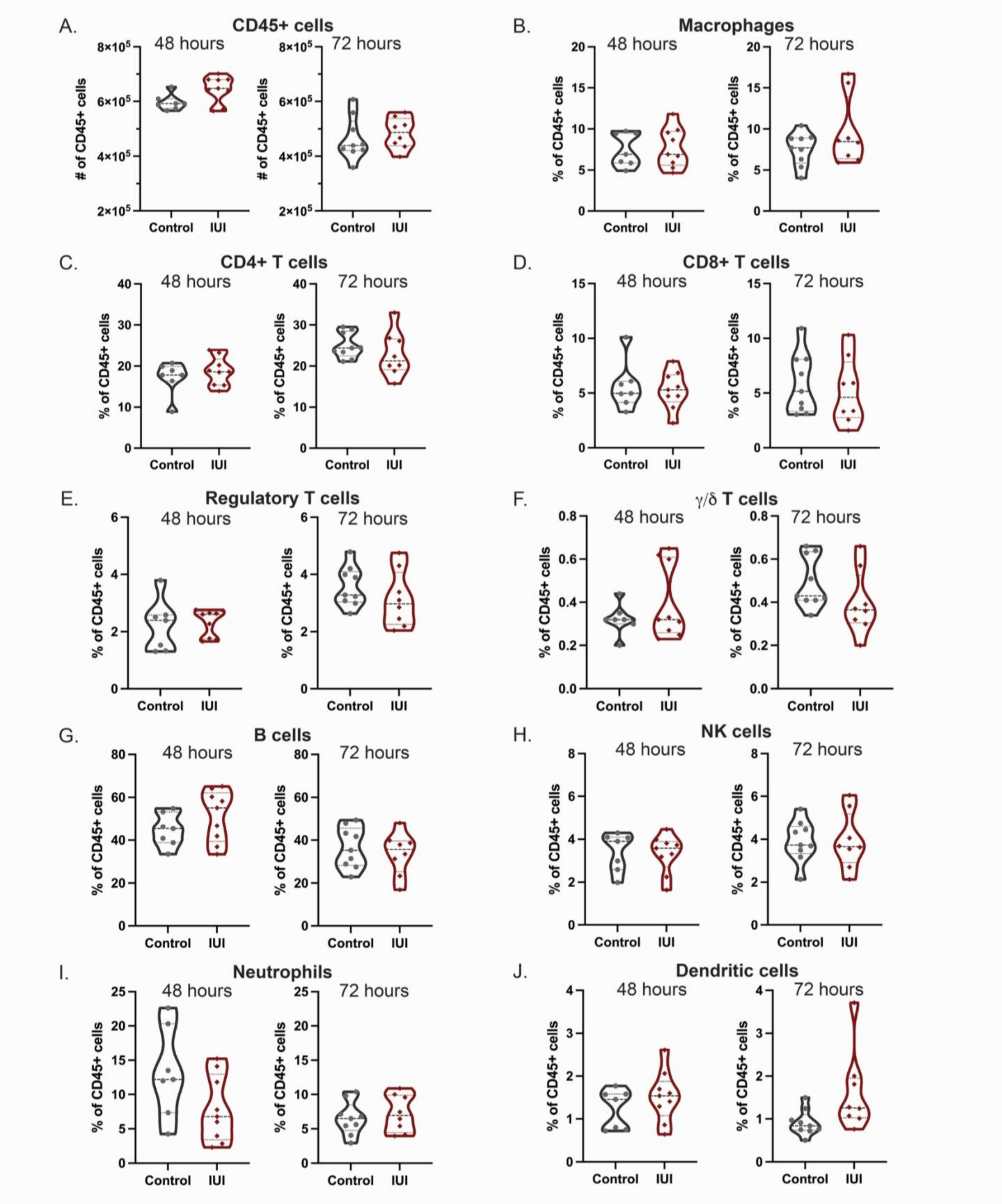

A.
CD45+ cells
48 hours
72 hours
# of CD45+ cells
Control
IUI
B.
Macrophages
% of CD45+ cells
C.
CD4+ T cells
D.
CD8+ T cells
E.
Regulatory T cells
F.
γ/δ T cells
G.
B cells
H.
NK cells
I.
Neutrophils
J.
Dendritic cells

Figure S2: Intrauterine inflammation does not alter systemic maternal immune populations. Maternal spleens were harvested at 48 and 72 hours post-uterine injection and immune populations were identified by flow cytometry. 10^6 events from each spleen sample were run on the flow cytometer. IUI had no significant effect on the maternal splenic immune populations examined, including: (A) CD45+ cell count, (B) macrophages, (C) CD4+ T cells, (D) CD8+ T cells, (E) regulatory T cells, (F) γ/δ T cells, (G) B cells, (H) NK cells, (I) neutrophils, and (J) dendritic cells.

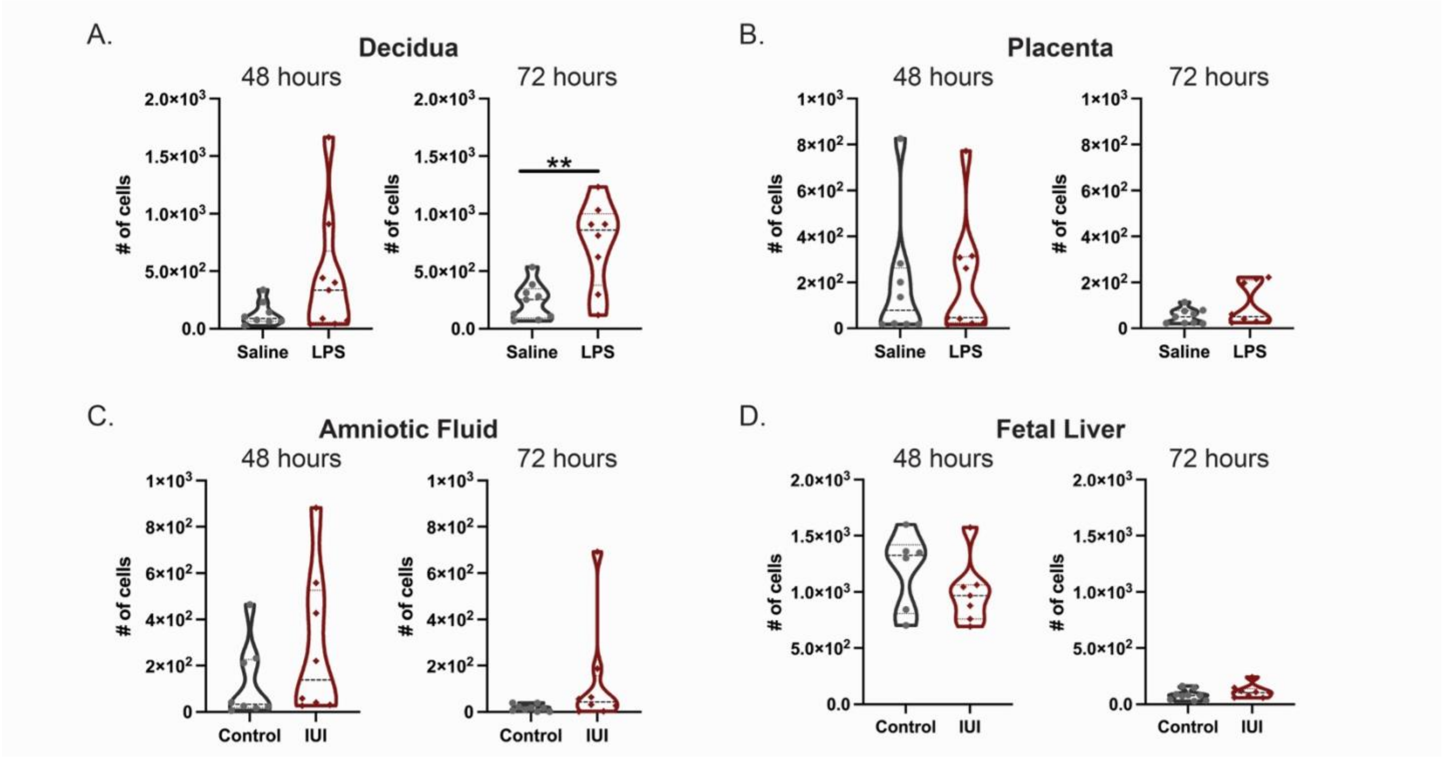

Figure S3: Decidual γ/δ T cells were increased 72 hours post-exposure to intrauterine inflammation. Decidua, placenta, amniotic fluid, and fetal liver were harvested at 48 and 72 hours post uterine injection and immune populations were analyzed by flow cytometry. Specifically, non-debris, single, live, CD45+, CD19-, CD4-, CD8-, Ly6G-, F4/80-, CD49b-, CD3+, γ/δ TCR+ cells were counted in each tissue: (A) decidua, (B) placenta, (C) amniotic fluid, and (D) fetal liver. All samples were analyzed by Shapiro-Wilk test for normality and F-test for unequal variance. Significance for decidual cells was determined by unpaired t-test with Welch's correction for unequal variance due to positive F-test. **:p<0.01.

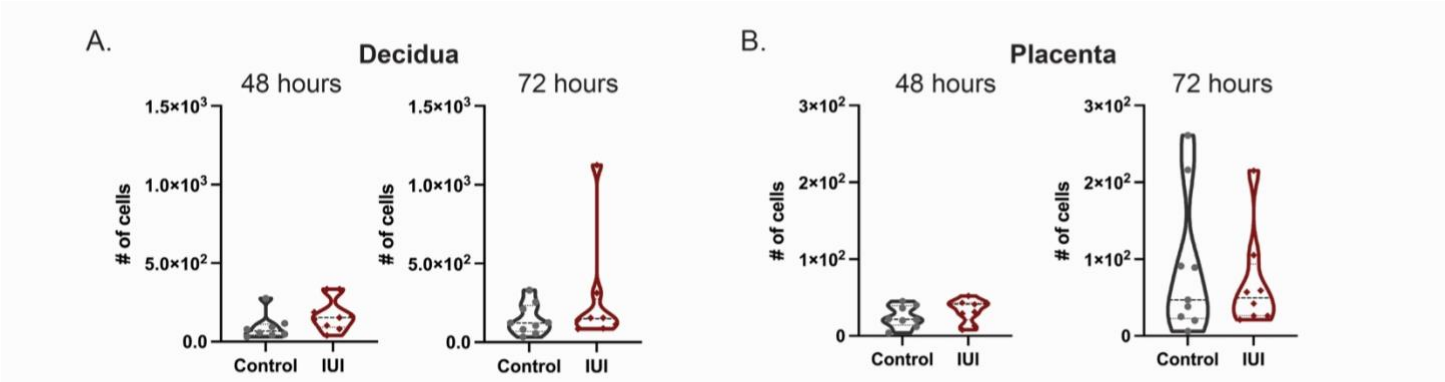

Figure S4: Intrauterine inflammation did not alter regulatory T cell numbers at the maternal-fetal interface. Decidua, placenta, amniotic fluid, and fetal liver were harvested at 48 and 72 hours post uterine injection and immune populations were analyzed by flow cytometry. Specifically, non-debris, single, live, CD45+, CD19-, CD8-, CD4+, FoxP3+ cells were evaluated. Few to no (<10 cells) Tregs were identified in the amniotic fluid or fetal livers. There was no difference in the number of Tregs in the (A) decidua or (B) placenta by exposure to intrauterine inflammation.
